# Mixed linage kinase 3 functions as a cGMP-dependent protein kinase I alpha substrate and regulates blood pressure and cardiac remodeling in vivo

**DOI:** 10.1186/2050-6511-16-S1-A24

**Published:** 2015-09-02

**Authors:** Timothy Calamaras, Robert Baumgartner, Guang-rong Wang, Roger Davis, Mark Aronovitz, David Kass, R Karas, Robert M Blanton

**Affiliations:** 1 Molecular Cardiology Research Institute, Tufts Medical Center, Boston, Massachusetts 02111, USA; 2 Program in Molecular Medicine, University of Massachusetts Medical School, Worcester, Massachusetts 01605, USA; 3 Department of Cardiology, Johns Hopkins Medical Institutions, Baltimore, Maryland 21205, USA

## 

Protein kinase G I alpha (PKGIα) counteracts hypertension and pathologic cardiac remodeling. These effects require the PKGIα leucine zipper (LZ) protein binding domain. However, PKGIα LZ-binding substrates mediating these effects remain incompletely understood. We previously demonstrated that Mixed Lineage Kinase 3 (MLK3) binds the PKGIα LZ domain in the heart. In the present study we hypothesized that MLK3 functions as a PKGIα substrate and cardiovascular effector.

We observed that recombinant MLK3 precipitated with affinity purified PKGIα but not with LZ mutant PKGIα. When PKGIα was precipitated with RP-cGMP beads, which inhibit PKG kinase activity, we observed decreased PKGIα-MLK3 co-precipitation, supporting a requirement of PKGIα kinase activity for MLK3-PKGIa interaction. PKGIα phosphorylated MLK3 in vitro as assayed by Western blot.

We next analysed mice with genetic deletion of MLK3. In the baseline state, MLK3^-/-^ mice display normal cardiac function as assessed by echocardiography and invasive cardiac hemodynamics. MLK3^-/-^ mice develop cardiac hypertrophy by 3 months of age (heart weight/tibia length 64.4 ± 1.9 mg/cm WT, 73.6 ± 2.1 mg/cm MLK3^-/-^; p<0.001; n=11 WT, 14 MLK3-/-). Compared with WT littermates, anesthetized MLK3^-/-^ mice have elevated blood pressure (BP) (94.3 ± 2.1 mmHg WT, 109.3 ± 2.5 mmHg MLK3^-/-^; p<0.001). Conscious male MLK3^-/-^ mice monitored continuously with implantable arterial radiotelemetry (10-12 weeks of age) had overt hypertension compared with WT littermates (Systolic BP: WT 121.5 ± 2.0 mmHg, MLK3^-/-^ 161.6 ± 5.1 mmHg; p<0.01; Diastolic BP: WT 87.0 ± 2.9 mmHg, MLK3^-/-^ 114.5 ± 2.7 mmHg; p<0.001; n=4 WT, 3 MLK3^-/-^). We observed no difference in baseline heart rate between genotypes.

Chronic administration of hydralazine (250 mg/L) normalized BP in MLK3^-/-^ mice, but did not completely inhibit cardiac hypertrophy. Further, in response to LV pressure overload by transaortic constriction (TAC), which equalized left ventricular (LV) systolic pressure between genotypes, MLK3^-/-^ mice had increased LV hypertrophy (LV/Tibia length) as well as elevated LV end diastolic pressure, and worsening of LV ejection fraction, preload recruitable stroke work, and other LV systolic and diastolic indices (n=8-10), indicating advanced cardiac dysfunction.

Together, our findings identify MLK3 as a direct PKGI substrate, and reveal that deletion of MLK3 leads to hypertension and pathologic cardiac hypertrophy. These findings support a model in which, in response to activation by PKGIα, MLK3 inhibits hypertension and cardiac hypertrophy. We conclude that identifying novel PKGIα LZ substrates, like MLK3, may reveal new candidate therapeutic targets for hypertension and heart failure.

